# Practical Notes on Diagnosis and Treatment

**Published:** 1909-09-11

**Authors:** 


### Practical Notes on Diagnosis and Treatment.

### Mental Deficiency in Childhood.

Slight degrees of cretinism are much more common than is generally supposed, though they are not always of congenital origin. In these cases stunting and delayed growth are the most marked features, combined with mental dulness and apathy. It is quite justifiable to try the therapeutic effect of doses of thyroid extract as an aid to diagnosis.—*Dr. C. Paget Lapage.*

### Treatment of Asthma.

A cup of strong black coffee is a traditional remedy for asthma; its effect probably depends on the caffeine which it contains. Another useful drug is nitrate of ethyl. Pilocarpine sometimes succeeds when other remedies fail. This acts on the vessels and relieves spasm. Adrenalin though it has an exactly opposite action is yet sometimes useful. To prevent the recurrence of the attacks, a combination of bromide and iodide of potassium is excellent.—*Dr. Samuel West.*

### The Treatment of Uric Acid.

In my opinion the late Sir Wm. Roberts's simple prescription of half a drachm of bicarbonate of potash in a tumbler of water at bedtime, to stem the nightly acid tide, is, on the whole, one of the most useful recommendations, apart from tonics, cures at watering-places, and change of scene and air.—*Dr. Goodhart.*

### X-Rays in Graves's Disease.

In almost every case there is a sedative effect which is manifested very early in the treatment. In 90 per cent. the rate of the heart diminishes and the palpitation is relieved. In two-thirds there is a gain in weight. With regard to the goitre the results are less uniform. At first it may become rather more tense, but gradually it softens, and in quite 20 per cent. of the cases it diminishes materially in size. Exophthalmos is the last symptom to yield to treatment, and in many cases it persists.—*Dr. A. C. Jordan.*

